# Supplementary figures and images for: Strongyloidiasis in Auckland: A ten-year retrospective study of diagnosis, treatment and outcomes of a predominantly Polynesian and Fijian migrant cohort
Source: PLoS Negl Trop Dis. 2024 Mar 28;18(3):e0012045. doi: 10.1371/journal.pntd.0012045 (PMC11003684; doi:10.1371/journal.pntd.0012045)

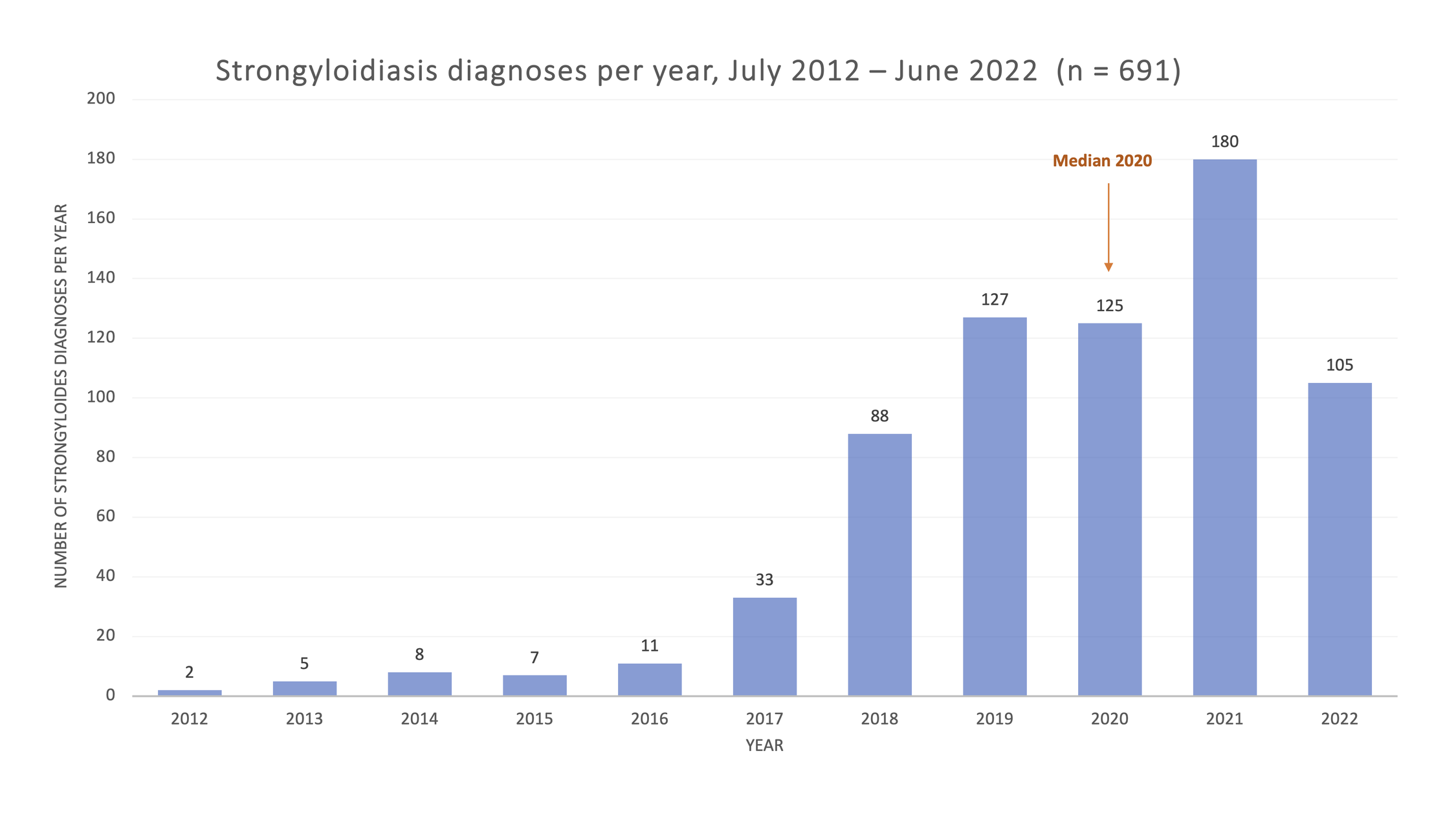

Supplement: S1 Fig — * Strongyloides serology testing became available in Aotearoa New Zealand in 2017 at Canterbury Health Laboratories (CHL). The total numbers of Strongyloides serology tests for the Auckland region at CHL were: 78 (2017), 331 (2018), 445 (2019), 515 (2020), 720 (2021), 524 (2022, to June). (TIF) [file pntd.0012045.s001.tif]
