# Supplementary material for: Strongyloidiasis in Auckland: A ten-year retrospective study of diagnosis, treatment and outcomes of a predominantly Polynesian and Fijian migrant cohort
Source: PLoS Negl Trop Dis. 2024 Mar 28;18(3):e0012045. doi: 10.1371/journal.pntd.0012045 (PMC11003684; doi:10.1371/journal.pntd.0012045)
Supplement: S1 Text — Table A. Definitions of medication-associated immunosuppression. Table B. Comparison of pre-treatment characteristics of strongyloidiasis cohort by ‘positive’ or ‘equivocal’ diagnosis (n = 691). 1 Other Polynesia: Niue Island (4), Tuvalu (4), American Samoa (2), Tahiti (1). 2 Other Pacific Island Countries and Territories (PICT): Kiribati (1). 3 Wilcoxon rank sum test (age); Fisher’s Exact test (other variables). (DOCX) [file pntd.0012045.s002.docx]

***Table A. Definitions of medication-associated immunosuppression***

| Evidence of pharmacy dispensing, or clinical documentation of prescription of any of the following within 1 month of diagnosis with strongyloidiasis: | |
| --- | --- |
|  | - Systemic corticosteroid (daily use of 10mg or more prednisone equivalent, *or* >20mg prednisone equivalent for >50% of month; i.e. 2mg of dexamethasone, 40mg hydrocortisone, 8mg methylprednisone) |
|  | - Antimetabolite agents (methotrexate, leflunomide, mycophenolate, azathioprine, cyclosporin, tacrolimus) |
|  | - Anti-IL4 or anti IL5 treatment (mepolizumab, benralizumab) |
|  | - TNF-alpha inhibitor (adalimumab, infliximab, etanercept) |
|  | - Other immunocompromising treatment as determined by clinical review of records by an Infectious Diseases Specialist, if under the care of oncology, neurology (multiple sclerosis), dermatology (psoriasis) |

***Table B. Comparison of pre-treatment characteristics of strongyloidiasis cohort by ‘positive’ or ‘equivocal’ diagnosis (n=691)***

|  | **Total (n= 691)** | **Positive (n=584)** | **Equivocal (n=107)** | **p-value^3^** |
| --- | --- | --- | --- | --- |
| **Age (years),** median (range) | 63 (15, 92) | 64 (15, 92) | 54 (15, 90) | <0.01 |
| **Male**  n (%) | 500 (72%) | 440 (75%) | 60 (56%) | <0.01 |
| **Region of birth** n (%) |  |  |  | <0.01 |
| Pacific Island Countries and Territories (PICT) | 481 (70%) | 422 (72%) | 59 (55%) |  |
| Polynesia | 350 (50%) | 301 (52%) | 49 (46%) |  |
| Samoa | 246 (36%) | 221 (38%) | 25 (24%) |  |
| Tonga | 55 (8%) | 44 (8%) | 11 (10%) |  |
| Cook Islands | 38 (6%) | 29 (5%) | 9 (8%) |  |
| Other Polynesia^1^ | 11 (2%) | 7 (1%) | 4 (4%) |  |
| Fiji | 130 (19%) | 120 (21%) | 10 (9%) |  |
| Other PICT^2^ | 1 (0.1%) | 1 (0.2%) | 0 (0%) |  |
| Aotearoa New Zealand | 88 (13%) | 66 (11%) | 22 (21%) |  |
| Asia | 77 (11%) | 60 (10%) | 17 (16%) |  |
| Southeast Asia | 33 (5%) | 30 (5%) | 3 (3%) |  |
| Indian Subcontinent | 24 (4%) | 17 (3%) | 7 (7%) |  |
| Other Asian Country | 20 (3%) | 13 (2%) | 7 (7%) |  |
| Africa | 10 (1%) | 6 (1%) | 4 (4%) |  |
| Other | 13 (2%) | 9 (2%) | 4 (4%) |  |
| Not available | 22 (3%) | 21 (4%) | 1 (1%) |  |
| **Ethnicity** n (%) |  |  |  | <0.01 |
| NZ Māori | 29 (4%) | 20 (3%) | 9 (8%) |  |
| Other | 203 (29%) | 161 (28%) | 42 (39%) |  |
| Pasifika | 459 (66%) | 403 (69%) | 56 (52%) |  |
| **District Health Board** n (%) |  |  |  | >0.9 |
| CMDHB | 347 (50%) | 292 (50%) | 55 (51%) |  |
| ADHB | 205 (30%) | 173 (30%) | 32 (30% |  |
| WDHB | 139 (20%) | 119 (20%) | 20 (19%) |  |
| **Immunocompromised** | 68 (10%) | 58 (10%) | 10 (10%) | >0.9 |
| **Diabetes** | 381 (55%) | 324 (55%) | 57 (53%) | 0.7 |
| **Alcohol dependence** | 25 (4%) | 23 (4%) | 2 (2%) | 0.4 |
| **Corticosteroid use within 6 months** | 188 (27%) | 153 (26%) | 35 (33%) | 0.2 |
| **HTLV-1** |  |  |  | >0.9 |
| Negative | 4 (1%) | 4 (1%) | 0 (0%) |  |
| Not tested | 687 (99%) | 580 (99%) | 107 (100%) |  |

^1^ Other Polynesia: Niue Island (4), Tuvalu (4), American Samoa (2), Tahiti (1)

^2^ Other Pacific Island Countries and Territories (PICT): Kiribati (1)

^3^ Wilcoxon rank sum test (age); Fisher’s Exact test (other variables)
