# Supplementary material for: Strongyloidiasis in Auckland: A ten-year retrospective study of diagnosis, treatment and outcomes of a predominantly Polynesian and Fijian migrant cohort
Source: PLoS Negl Trop Dis. 2024 Mar 28;18(3):e0012045. doi: 10.1371/journal.pntd.0012045 (PMC11003684; doi:10.1371/journal.pntd.0012045)
Supplement: Attachment — Submitted filename: StrongAuckland_PLOS NTD Response to reviewers.docx [file pntd.0012045.s003.docx]

**Response to comments by reviewers of manuscript submitted to PLOS NTD :** *Strongyloidiasis in Auckland: a ten-year retrospective study of diagnosis, treatment and outcomes of a predominantly Polynesian and Fijian migrant cohort*

The authors are grateful for the carefully considered, constructive comments made by the reviewers of this manuscript. We agree that the review process has improved the manuscript, and have detailed our responses to specific comments below.

**--------------------------------------------------------------------------------------------------------------------------------------**

**Responses to reviewer 1:**

***1.    Line 157-162: readers would be interested in knowing how representative the study population is; authors should report the coverage of the population by the datasets (do they capture all persons seen in any clinic/hospital in the Auckland region, or just part of them?).***

Thank you for highlighting this point: yes, the two laboratories (LabTests and LabPLUS) were responsible for processing all parasitology specimens within the Auckland region during the study period. To improve clarity, we have added *‘all’* to line 165 of the revised manuscript.

***2.    Line 164-171: I wonder if authors can provide information on the indication of serological testing. Pre-test probability would be different between patients who were tested for clinical concerns, compared with those tested for screening purposes without clinical suspicion.***

Thank you for raising this point: we wholeheartedly agree with your statement. However unfortunately we did not collect the indication for testing, due to concerns about the reliability of retrospective determination of indication from documentation in clinical records.

To highlight this limitation, we have inserted a sentence into the discussion (lines 414-417 of the revised manuscript):

“*Secondly, as the indication for S. stercoralis testing was not collected, the seropositivity rates represent overall practice in Auckland during the study period, and could be expected to vary between clinical scenarios with different pre-test probabilities.*”

***3.    Line 186-188: I can understand the authors’ rationale here, but I am not certain if “equivocal” cases should be treated in the same way as others, as inclusion of these patients can skew some of analyses. Perhaps clearer distinction should be made between positive and equivocal cases, although authors try to do so to some extent in the manuscript. For example, it is probably not always wrong to not treat cases with equivocal results, whereas no treatment for positive cases is probably not good.***

Thank you for this observation. We agree that, in the absence of other diagnostic evidence or immunocompromise, ‘equivocal’ serology does not provide compelling evidence to support treatment in some people. However, there were far fewer ‘equivocal’ diagnoses than ‘positive’ (107 vs. 584), and only 37 people with ‘equivocal’ diagnoses (compared with 447 ‘positive’) received treatment. Hence although we do not believe that differences between the ‘equivocal’ and ‘positive’ groups are likely to alter the main outcome of this study, we agree it is reasonable to include an additional post-hoc analysis comparing the ‘positive’ and ‘equivocal’ groups.

Although we defer to editorial decision, our preference is that this post-hoc comparison be added as an additional supplementary table (*Table S2*) rather than replacing Table 1, and accompanied by the following additional sentences in the methods and results sections (lines 259-261, and lines 276-280 of the revised manuscript) :

*“A second post-hoc analysis compared demographic and clinical variables between people with ‘positive’ and ‘equivocal’ diagnoses, using the Wilcoxon rank sum test to assess the variable ‘age’, and Fisher’s exact test for other variables.”*

*“Participants with ‘equivocal’ diagnoses had similar rates of comorbidity to those with ‘positive’ diagnoses, but were less likely to be born in PICT (55% vs 72%), had a younger mean age (54 vs 64 years) and a lower proportion were male (56% vs. 75%)*(Table S2).”

***Table S2: Comparison of pre-treatment characteristics of strongyloidiasis cohort by ‘positive’ or ‘equivocal’ diagnosis (n=691)***

|  | **Total (n= 691)** | **Positive (n=584)** | **Equivocal (n=107)** | **p-value^3^** |
| --- | --- | --- | --- | --- |
| **Age (years),** median (range) | 63 (15, 92) | 64 (15, 92) | 54 (15, 90) | <0.01 |
| **Male**  n (%) | 500 (72%) | 440 (75%) | 60 (56%) | <0.01 |
| **Region of birth** n (%) |  |  |  | <0.01 |
| Pacific Island Countries and Territories (PICT) | 481 (70%) | 422 (72%) | 59 (55%) |  |
| Polynesia | 350 (50%) | 301 (52%) | 49 (46%) |  |
| Samoa | 246 (36%) | 221 (38%) | 25 (24%) |  |
| Tonga | 55 (8%) | 44 (8%) | 11 (10%) |  |
| Cook Islands | 38 (6%) | 29 (5%) | 9 (8%) |  |
| Other Polynesia^1^ | 11 (2%) | 7 (1%) | 4 (4%) |  |
| Fiji | 130 (19%) | 120 (21%) | 10 (9%) |  |
| Other PICT^2^ | 1 (0.1%) | 1 (0.2%) | 0 (0%) |  |
| Aotearoa New Zealand | 88 (13%) | 66 (11%) | 22 (21%) |  |
| Asia | 77 (11%) | 60 (10%) | 17 (16%) |  |
| Southeast Asia | 33 (5%) | 30 (5%) | 3 (3%) |  |
| Indian Subcontinent | 24 (4%) | 17 (3%) | 7 (7%) |  |
| Other Asian Country | 20 (3%) | 13 (2%) | 7 (7%) |  |
| Africa | 10 (1%) | 6 (1%) | 4 (4%) |  |
| Other | 13 (2%) | 9 (2%) | 4 (4%) |  |
| Not available | 22 (3%) | 21 (4%) | 1 (1%) |  |
| **Ethnicity** n (%) |  |  |  | <0.01 |
| NZ Māori | 29 (4%) | 20 (3%) | 9 (8%) |  |
| Other | 203 (29%) | 161 (28%) | 42 (39%) |  |
| Pasifika | 459 (66%) | 403 (69%) | 56 (52%) |  |
| **District Health Board** n (%) |  |  |  | >0.9 |
| CMDHB | 347 (50%) | 292 (50%) | 55 (51%) |  |
| ADHB | 205 (30%) | 173 (30%) | 32 (30% |  |
| WDHB | 139 (20%) | 119 (20%) | 20 (19%) |  |
| **Immunocompromised** | 68 (10%) | 58 (10%) | 10 (10%) | >0.9 |
| **Diabetes** | 381 (55%) | 324 (55%) | 57 (53%) | 0.7 |
| **Alcohol dependence** | 25 (4%) | 23 (4%) | 2 (2%) | 0.4 |
| **Corticosteroid use within 6 months** | 188 (27%) | 153 (26%) | 35 (33%) | 0.2 |
| **HTLV-1** |  |  |  | >0.9 |
| Negative | 4 (1%) | 4 (1%) | 0 (0%) |  |
| Not tested | 687 (99%) | 580 (99%) | 107 (100%) |  |

^1^ Other Polynesia: Niue Island (4), Tuvalu (4), American Samoa (2), Tahiti (1)

^2^ Other Pacific Island Countries and Territories (PICT): Kiribati (1)

^3^ Wilcoxon rank sum test (age); Fisher’s Exact test (other variables)

***4.    Line 193: define “DHB”.***

Our apologies for this oversight: ‘DHB’ refers to ‘District Health Board’ as defined in line 148 of the revised manuscript. We have added ‘DHB’ in parentheses in line 148 to clarify this acronym.

 ***5.    Line 202-204: how well does this approach capture ivermectin dispensing? Is there a possibility that availability of ivermectin could affect treatment, and providers could prescribe albendazole instead, even though it is less efficacious?***

Thank you for this question. As mentioned in lines 225-226 of the revised manuscript, ivermectin is the only publicly funded medication available for the treatment of strongyloidiasis in Aotearoa New Zealand, and requires ‘special authority’ from an Infection specialist prior to prescription. Alternative treatments (i.e. albendazole) also require ‘special authority’ approval from an Infection specialist, but are only approved for other indications.

Additionally, during data collection for this study, hospital medical records including medical charts were reviewed individually, and no other hospital-administered treatments for strongyloidiasis were identified.

The completeness of community dispensing data derived from ‘TestSafe’ has been previously assessed. In 2006, it was estimated that the pharmaceutical collection which feeds TestSafe accounts for 92% of all community prescriptions. This proportion is anticipated to have significantly increased since 2006, with subsequent improved linking to National Health Index (NHI – a national medical identification number), and an opt-out strategy for data reporting.^1^

While we cannot completely exclude privately funded prescriptions, we believe these would be extremely uncommon, and unlikely to meaningfully contribute to under detection of ivermectin treatment in this cohort.

^1^ Horsburgh, S., Malik, M., Noris, P., Harrison‐Woolrych, M., Tordoff, J.M., Becket, G., Herbison, P., Parkin, L., & Reith, D.M. (2009). Prescribing and dispensing data sources in New Zealand: their usage and future directions. Available at: <https://www.researchgate.net/publication/45637891_Prescribing_and_dispensing_data_sources_in_New_Zealand_their_usage_and_future_directions>

 

***6. Line 268: is there anything special about before and after 2019? This is not mentioned in Discussion. Is it because of higher prevalence of strongyloidiasis after 2017-2019, more immigrants in this time period, overall increased testing, or more testing is populations with higher prevalence? Readers would at least be interested in how many serological testing was done in each year.***

Thank you for this question. The likely explanation for the increase in diagnoses later in the study period is an increase in rates of *Strongyloides* serology testing after becoming available within Aotearoa New Zealand in 2017. We agree that including this information would be of interest to readers, and have added the following sentences to the revised manuscript (lines 280-282 in the results section, and lines 609-611 of *Figure S1*)

*“The Auckland region’s annual requests for Strongyloides serology increased from 78 in 2017, to 720 in 2020. Consequently, most participants were diagnosed after 2019 (405/691; Figure S1). “*

** Strongyloides serology testing became available in Aotearoa New Zealand in 2017 at Canterbury Health Laboratories (CHL). The total numbers of Strongyloides serology tests for the Auckland region at CHL were: 78 (2017), 331 (2018), 445 (2019), 515 (2020), 720 (2021), 524 (2022, to June)*

***7.    Line 329-337: how many of these patients were equivocal? As above in Methods #3, the populations with equivocal diagnosis and positive diagnosis are likely quite different, which is supported by the Line 335-337. These populations should likely be analyzed separately.***

Thank you for this question. We have added a general comparison between ‘equivocal’ and ‘positive’ groups to the manuscript, as described in our response to question 3, above. As described in lines 323-324 of the modified manuscript, and Figure 3, only 37 participants with ‘equivocal’ diagnosis received treatment, compared with 447 with ‘positive’ diagnosis. Due to the small numbers we do not feel that presenting a post-hoc analysis of treatment outcome between these subgroups is likely to add to the key messages of this study.

***8.    Line 344-346, and Discussion 384-385: note that this study is different from universal screening. Thus, the “positive” rate is different from seroprevalence. The significance of this data depends on pretest probability (ie as in Methods #2 above, whether there was clinical suspicion before testing).***

We agree with this statement. In addition to our response to point 2 (above), we attempt to acknowledge this point in lines 394-395 and 414-417 of the revised manuscript.

***9.    Line 357-365: this is technically a result, and should not appear for the first time in Discussion. It is currently rather anecdotal. Is there anyway authors could quantify this, or provide description in a way that is scientifically sound?***

We thank the reviewer for this suggestion. Unfortunately, this study was not designed to evaluate reasons for non-treatment. Lines 357-365 reflect hypotheses proffered by the authors to assist readers with understanding possible explanations for a lower than expected rate of treatment. These hypotheses are based on observations made during data collection, that while are somewhat anecdotal, reflect important considerations for development of a local strongyloidiasis guideline. For example, our first hypothesis “*a) abnormal serology results not being electronically communicated from laboratories to the responsible clinician*” has already led to identification of a systemic data linkage problem between laboratories.

However, we agree with the reviewer that the previous wording of this paragraph over-stated the strength of data informing our hypotheses, and have re-worded the paragraph accordingly (lines 372-379 of the revised manuscript:

*“..The low proportion of treated participants reported in this study might have been contributed to by under-ascertainment of ivermectin dispensing.* ***However, based on observations made during data collection, the following contributions are hypothesised to be more plausible explanations for undertreatment of this cohort:*** *a) abnormal serology results not being electronically communicated from laboratories to the responsible clinician, b) abnormal results not actioned by the requesting team, c) deferral of responsibility to arrange treatment to another clinician (e.g., primary care provider), and d) misplaced confidence in the negative predictive value of stool microscopy* ***These hypotheses might*** *suggest that improved clinician education and system-level interventions should be the focus of initial regional interventions to improve the management of strongyloidiasis.”*

***10.    Line 367-378: authors endorse test of cure strongly. It is employed in many studies to facilitate outcome assessment, but in clinical practice, as authors state, it is controversial. It is unclear how problematic this result is, and it is also unclear if this is important enough to be included in Abstract. Authors should consider evaluating clinical failure (though authors provide some anecdotes).***

We respectfully disagree with the reviewer’s statement that we ‘endorse test of cure strongly’ in lines 367-378 of the original manuscript. In this paragraph we highlight the low proportion of participants for whom we were able to evaluate a treatment outcome, acknowledge the uncertainty regarding the role of ‘test of cure’ for all people with strongyloidiasis, but suggest that for people with the highest risk of severe/relapsed strongyloidiasis, post-treatment ‘test of cure’ might be considered, in line with published guidance. As described, we provide anecdotes to provide support for such a stance.

With regards to considering evaluation of clinical failure (similar to response 2, above), unfortunately our retrospective study design, which was reliant on documentation by general clinicians, would not allow for determination of clinical symptoms prior to treatment, nor improvement of symptoms post-treatment. We would certainly be interested in conducting prospective evaluation of clinical success of treatment in a ‘high risk’ population in the future.

***11. To support the conclusion, I think equivocal vs positive cases should be separated. Limitations – see above in Methods and Results.***

Thank you for this suggestion. Please see responses to points 3 for additional analysis by ‘positive’ and ‘equivocal’ diagnoses. Similar to our response to point 7, given the low proportion of total cases with ‘equivocal’ diagnosis (107 / 691, 15%), we do not believe that re-stating the treatment rate for participants with ‘positive’ (77%) and ‘equivocal’ (35%) diagnoses, compared with considering the ‘total’ treatment rate (70%) adds meaningfully to the main outcome of this study. We feel that, particularly with the addition of our amendments in response to point 3, there is now sufficient delineation of the difference between these groups in other sections of the manuscript.

However, we agree that it would be clearer if we were to emphasise the treatment rate of 70% applies to the ‘total’ cohort, and we have added this to lines 453 and 74 of the conclusion and abstract respectively.

***12. Consider making Introduction more concise by providing information that is necessary to set the stage for the study only.***

Thank you for this suggestion. Although we agree that a more fulsome introduction is wholly unnecessary for readers among the Neglected Tropical Diseases community, the authors hope that this paper will also be read by a generalist clinical audience in Aotearoa New Zealand, Polynesia and Fiji, many of whom may be unfamiliar with *Strongyloides stercoralis.* As such, with editorial permission, we would prefer to keep the introduction in its current state. *--------------------------------------------------------------------------------------------------------------------------------------*
**Responses to reviewer 2**

***1. Minor modification: please check table 3 last column on negative tests. total is 1,963, but adds up to 1,965. Other countries shows 152 total in description, with 155 total in row in table. (The PICT, Other Asian and African countries match the table).***

Thank you very much for your careful review of our manuscript resulting in identification of this error, which is likely to have been made during revision of table 3 late in the drafting process. The correct number of people born in ‘other’ countries with ‘negative’ serology should be 140 (rather than 142). We have corrected this error in the revised manuscript.

*--------------------------------------------------------------------------------------------------------------------------------------*

**Responses to reviewer 3:**

***1. Line 146 – Were there any differences in the quality of data (and conclusions drawn) from the three distinct healthcare providers mentioned?  Was there any evidence of bias between them?***

Thank you for this question. Although we did not aim to specifically assess differences in practice or outcomes between the three district health boards (DHBs), we did not expect to encounter, nor did we observe any clear differences in data quality or evidence of bias between the three DHBs.

We agree that inter-hospital variation will be an important issue to consider when we evaluate adherence to planned regional guidance for the evaluation and management of strongyloidiasis, once completed.

***2. Line 167-9 – Since serological testing was the sole diagnostic methodology In a large proportion of the cohort (64%) it would be useful to provide the accepted sensitivity, specificity and accuracy of the assays at the selected cutoff OD (in addition to reference to the previous publications).***

Thank you for this comment. We are happy to include diagnostic sensitivity and specificity included by the manufacturer. The reported performance of the BORDIER and similar ELISA IgG assays varies widely between different populations, and we are unaware of any study exploring performance in a population similar to Auckland, New Zealand. We have therefore amended line 173-175 of the revised manuscript to the following:

*“The estimated performance characteristics of this assay as reported by the manufacturer include a diagnostic sensitivity of 90% and specificity of 70-90%. Other estimates vary and are described elsewhere [20,21].”*

***3. Line 234 – A 50% reduction in OD in the ELISA test was defined as a serological response.  Why was this level of changed selected? The decrease in OD is notoriously variable, unlike a change in titres of antibodies, is variable and, for example, in the in-house assay used by WestPath, defined as positive for OD>0.25 (***[***https://doi.org/10.1371/journal.pntd.00056070***](https://doi.org/10.1371/journal.pntd.00056070)***)***

We agree with your comment that defining a reduction in OD as a marker of treatment response is imperfect. To ensure our results were comparable to other recently published trials, we adopted the definition of serologic response (for the BORDIER assay) described by the ‘Strong Treat 1-4’ trial. We welcome ongoing research into the optimal use of serology to assess treatment response, particularly in the Pacific.

With regrets, we are unable to comment on the paper you have suggested as the link appears to be broken.

***4. Line 311 – What doses of ivermectin were used in the cases of severe strongyloidiasis?***

The daily dose of ivermectin used for treatment of severe strongyloidiasis (range 12-30mg) did not differ from non-severe cases (range 3-33mg). However in general, daily treatment was administered until symptom resolution and/or negative stool microscopy was obtained.

***5. Line 314 – Post-treatment “test of cure” serology was performed in only 20% of cases.  How was the outcome of treatment determined in the remaining cases?***

We had anticipated a low proportion of the study cohort would have post-treatment serology. As such, we also considered resolution of eosinophilia and / or resolution of microscopically confirmed strongyloidiasis when determining response to treatment. This is described in ‘definitions of treatment outcome’ in the methods section (lines 234-248 of the revised manuscript). As described in lines 333-336 of the revised manuscript, despite considering serology, eosinophilia and stool microscopy, we were could not determine the outcome of treatment in 49% of individuals.

***6. Line 474 – In Figure 2, the row totals for serology positive and negative subgroups should be added to the Eosinophilia column***

Thank you for highlighting this point of potential confusion. As such, we have adjusted the formatting in the ‘total’ columns in Figure 2 to more clearly demonstrate that ‘total’ include rows with and without eosinophilia. Although we would be happy to add a subtotal column to the Figure if requested by the reviewer and editorial team, we feel that the current formatting summarises the data in a way that is likely to be easier for readers to quickly capture the most important data presented.
